# When Do Tumours Develop? Neoplastic Processes Across Different Timescales: Age, Season and Round the Circadian Clock

**DOI:** 10.1111/eva.70024

**Published:** 2024-10-22

**Authors:** Margaux Bieuville, Antoine M. Dujon, Nynke Raven, Beata Ujvari, Pascal Pujol, Zahra Eslami‐S, Catherine Alix Panabières, Jean‐Pascal Capp, Frédéric Thomas

**Affiliations:** ^1^ CREEC (CREES), Unité Mixte de Recherches IRD 224‐CNRS 5290‐Université de Montpellier Montpellier France; ^2^ Institute of Organismic and Molecular Evolution (iomE) Johannes Gutenberg‐Universität Mainz Germany; ^3^ Institute for Quantitative and Computational Biosciences (IQCB) Johannes Gutenberg‐Universität Mainz Germany; ^4^ School of Life and Environmental Sciences Deakin University Waurn Ponds Victoria Australia; ^5^ Oncogenetic Department University Medical Centre of Montpellier Montpellier France; ^6^ Laboratory of Rare Human Circulating Cells and Liquid Biopsy (LCCRH) University Medical Centre of Montpellier Montpellier France; ^7^ European Liquid Biopsy Society (ELBS) Hamburg Germany; ^8^ Toulouse Biotechnology Institute University of Toulouse, INSA, CNRS, INRAE Toulouse France

**Keywords:** age, circadian rhythm, evolution, neoplasia risk, season, senescence

## Abstract

While it is recognised that most, if not all, multicellular organisms harbour neoplastic processes within their bodies, the timing of when these undesirable cell proliferations are most likely to occur and progress throughout the organism's lifetime remains only partially documented. Due to the different mechanisms implicated in tumourigenesis, it is highly unlikely that this probability remains constant at all times and stages of life. In this article, we summarise what is known about this variation, considering the roles of age, season and circadian rhythm. While most studies requiring that level of detail be done on humans, we also review available evidence in other animal species. For each of these timescales, we identify mechanisms or biological functions shaping the variation. When possible, we show that evolutionary processes likely played a role, either directly to regulate the cancer risk or indirectly through trade‐offs. We find that neoplastic risk varies with age in a more complex way than predicted by early epidemiological models: rather than resulting from mutations alone, tumour development is dictated by tissue‐ and age‐specific processes. Similarly, the seasonal cycle can be associated with risk variation in some species with life‐history events such as sexual competition or mating being timed according to the season. Lastly, we show that the circadian cycle influences tumourigenesis in physiological, pathological and therapeutic contexts. We also highlight two biological functions at the core of these variations across our three timescales: immunity and metabolism. Finally, we show that our understanding of the entanglement between tumourigenic processes and biological cycles is constrained by the limited number of species for which we have extensive data. Improving our knowledge of the periods of vulnerability to the onset and/or progression of (malignant) tumours is a key issue that deserves further investigation, as it is key to successful cancer prevention strategies.

## 1 Introduction

Neoplasia, defined as an abnormal tissue growth (malignant or not), is a pathology that appeared at the end of the Precambrian period (about a billion years ago) with the appearance of multicellular organisms (Domazet‐Loso and Tautz 2010; Ujvari, Roche, and Thomas 2017). It therefore concerns all metazoans, being present in animals from hydras to whales (Domazet‐Lošo et al. 2014; Aktipis et al. 2015; Vincze et al. 2022). Not only are neoplastic processes ubiquitous in metazoans, but they are also present, sooner or later, in the bodies of all individuals of these species (Thomas et al. 2018). They may be more or less abundant, more or less aggressive (the most serious form is metastatic cancer, which is usually fatal, Dillekås, Rogers, and Straume 2019), but in all cases, they are omnipresent (Folkman and Kalluri 2004; Maley et al. 2017). Moreover, when neoplasia progresses to a malignant invasive tumour (i.e. cancer), the associated morbidity and mortality could represent an evolutionary pressure for species as physiological functions (such as foraging or reproduction) and survival of individuals are compromised (see Dujon et al. 2022 for a systematic review of the age‐specific link between reproduction and cancer risk). Therefore, studying defence mechanisms against the neoplastic processes in different species can help us understand what shapes the risk variation between species (Nunney et al. 2015).

The search for such mechanistic explanations has shed light on numerous factors that influence the risk of developing cancer from genes and cellular processes (Hanahan and Weinberg 2011), to life‐history traits (e.g. mammalian litter size in Boddy et al. 2020 and in Dujon et al. 2023; avian developmental rates in Møller, Erritzøe, and Soler 2017), diet (Vincze et al. 2022) and its associated microbiome (Kapsetaki et al. 2022), metabolism (Dang 2015) or lifespan and body size (Boddy et al. 2020; Vincze et al. 2022). Extrinsic factors such as exposure to pathogens (Ewald and Swain Ewald 2015) or environmental degradation due to pollution (Dujon et al. 2021) have also been shown to shape neoplastic processes both within and between species.

What remains imperfectly understood, however, is the precise temporal dynamics of these processes over the life course of an individual and their age‐specific mechanisms. Given the aforementioned examples, it is unlikely that the probability of the onset and/or dynamics of neoplastic processes is strictly the same at all times. In other words, some periods of life are more favourable to oncogenesis than others.

Identifying the periods during which neoplasia is initiated and progresses (possibly towards an invasive malignant—i.e. cancerous—stage), and understanding the causal mechanisms involved, would be of great use in cancer prevention as it would help to tailor prophylactic and therapeutic approaches. In the following, we use the word ‘cancer’ when papers cited refer to clinically diagnosed cancer or invasive malignant tumour and ‘neoplasia’ otherwise. Considering both evolutionary and proximal explanations, we present here a synthesis of these issues, considering recent advances in knowledge about the dynamics of neoplastic processes as a function of age, seasons and time of day (i.e. circadian rhythm). Most of the studies with the required level of detail are done in humans and lab/domesticated species. Nonetheless, when available, we include works done in a broader range of species.

### 1.1 Neoplasia and Cancer Risk as a Function of Age

In humans, age is shown as the biggest overall risk factor and a risk factor in individual tumour types as well (cancer fact sheet). Consequently, the cancer risk (both in incidence and mortality) varies with age. While data are scarce for other species (but see below), one could hypothesise that—depending on whether tumour‐promoting mechanisms are shared—other animal species will display a (dis)similar age‐specific pattern. In this section, we will look at the association between age and cancer risk along three axes: the association of age (i) with cancer incidence, (ii) with the distribution of cancer types, and (iii) with cancer prognosis and mortality. We then finish with a section summarising the information available on other species than humans.

#### 1.1.1 Age and Cancer Incidence

With age being a major risk factor in humans and with ageing populations being associated with an increasing cancer prevalence (De Angelis et al. 2024), cancer is often considered a disease of ageing. Early epidemiological models of carcinogenesis inferred from age‐specific incidence curves showed that 6–7 discrete mutations were necessary for the onset of various tumours (coined the multistage model of carcinogenesis, Armitage and Doll 1954) and therefore showed the power relation between cancer incidence and age. This mutation‐centric view of carcinogenesis has initially found support. For instance, Tomasetti and Vogelstein demonstrated that the lifetime risk of getting cancer in 31 organs was correlated with the lifetime number of stem cell divisions in those tissues (Tomasetti and Vogelstein 2015). Subsequent works have shown that some of their assumptions might bias their results (Rozhok, Wahl, and DeGregori 2015). Furthermore, more complex models accounting for selection and clonal expansion of lineages fit incidence data more closely (Nunney and Thai 2020). Other works (and see below) also pointed out the role of the environment and its age‐specific changes with the idea of a ‘permissive window’ for tumourigenic processes (Sieber, Tomlinson, and Tomlinson 2005).

The idea that cancer and aging go hand in hand does not come from the multistage model alone but also from the fact that tumourigenesis and senescence (defined here as the decrease in physiological functions associated with increasing age) share common mechanisms (e.g. loss of proteostasis or epigenetic alterations, López‐Otín et al. 2013; Finkel, Serrano, and Blasco 2007). Nonetheless, this view fails to (i) account for important evolutionary processes shaping carcinogenic processes and (ii) explain two features of cancer epidemiology in humans (and their non‐human counterparts).

##### 1.1.1.1 Cancer is Not Only a Disease of Adulthood

To start with, while increasing age is a risk factor in humans, cancer is also found in children (Force et al. 2019). As some adult cancers have been shown to develop from a limited number of mutations (e.g. Tomasetti et al. 2015), it has been hypothesised that paediatric cancer might be the result of ‘bad luck’: paediatric cancer would be similar to adult cancer, except for the exceptional speed of mutation accumulation. This hypothesis is partly supported for two reasons. On the one hand, paediatric cancers are relatively rare compared to adult cancers because the probability of accumulating enough driver mutations is lower (e.g. UK data for the years 2016–2018, Cancer Research Organisation). On the other hand, it has been shown that some paediatric cancers result from a reduced number of mutations (Lawrence et al. 2013; Vogelstein et al. 2013).

However, other features of paediatric cancers highlight the importance of considering them in a different disease category. First, the distribution of cancer types among paediatric tumours differs from that in adults (see below). Second, the types of (epi)genetic alterations found in paediatric tumours are different from those in adults. For instance, ‘catastrophic’ events such as chromothripsis (Ma et al. 2018) and a high frequency of mutations in genes involved in epigenetic regulation (Huether et al. 2014) are found to drive paediatric tumourigenesis. Third—and this argument is also relevant for adult tumours following more closely the multistage predictions—a substantial part of the mutation load is thought to accumulate during embryonic development (DeGregori 2013). This suggests mutation accumulation is not the only driver: a change in the cellular adaptive landscape is necessary for those mutated cells to progress towards malignancy (see evidence reviewed in Rozhok and DeGregori 2015). This has for instance been found in some cases of acute lymphoid leukaemia (Gale et al. 1997). Finally, the relationship between age‐specific cancer incidence during childhood and age is not increasing monotonically (as one would expect if incidence was strictly a function of the waiting time to accumulate enough mutations). Indeed, children between 0 and 4 and between 15 and 19 are more at risk than children between the ages of 5 and 14 (Siegel et al. 2023). Even within a subgroup of tumours, age‐specific incidence curves can drastically differ. For instance, acute lymphoblastic leukaemia (ALL) peaks around the age of 4 years in children while the acute myeloid leukaemia (AML) incidence rate is rather constant across all childhood age categories (Marcotte et al. 2021).

Overall, in this section, we demonstrated that the multistage model does not hold for paediatric tumours as they do not exhibit the expected power law relationship between age and cancer risk.

##### 1.1.1.2 Cancer Incidence in Adulthood

During adulthood (defined here as the time after sexual maturity and, in determinate growth species, after growth), the relationship between age and cancer risk depends on the species considered.

###### 1.1.1.2.1 In Humans and Domestic Species: Increase in Incidence Rates With Age

In humans, cancer incidence rates increase with age (Harding, Pompei, and Wilson 2011; Thakkar, Villano, and McCarthy 2014; Cook et al. 2009) up to a certain point (see below). This relation stays true when all tumours are pooled together and for individual tumour types. In domestic dogs and cats, a similar pattern has been found in different studies (Dobson et al. 2002; Vascellari et al. 2009; Rafalko et al. 2023). Older datasets show that domestic bovine and equine species also display an age‐dependent cancer incidence (Priester and Mantel 1971).

It has been reviewed elsewhere how the domestication process could influence the risk of developing cancer compared to wild species (Thomas et al. 2020) and it has been proposed that humans and domestic dogs display a high cancer prevalence because of a recent evolutionary extension of their lifespan (Sarver et al. 2022). These works highlight the fact that age‐specific cancer incidences are the results of multiple evolutionary processes that are important to acknowledge when presenting results in comparative oncology. Nonetheless, some non‐domestic model organisms show that the multistage model is supported even in the absence of a recent longevity extension. For instance, in the short‐lived fish *Nothobranchius furzeri*, it has been shown that (i) incidence of neoplasia does increase with age and (ii) increasing age is associated with a progression towards higher degrees of malignancy (Di Cicco et al. 2011).

From an evolutionary perspective, it is interesting to note that the human cancer incidence rate starts to significantly increase late in adulthood (data from Cancer Research UK 2021) and that the median age at diagnosis is around 65 years old (all sites pooled) (data from the National Cancer Institute 2021). This suggests that protective mechanisms evolved so that the onset of tumour would start late in the reproductive period. While age‐specific estimates are rare in other species, this is congruent with works showing that large size or extended longevity are associated with the evolution of protective mechanisms in different species (Schraverus, Larondelle, and Page 2022).

###### 1.1.1.2.2 Strains, Carcinogen Exposure and Age Have Complex Associations With Cancer Incidence in Laboratory Rodents

Other non‐domestic model organisms add complexity to the previous section. Indeed, in humans, the log–log relationship between cancer incidence and age is linear, indicating a power law relationship (age being the power exponent). In an early study, Peto et al. (1975) tested whether the same feature could be found in laboratory mice and showed that exposure duration to a carcinogen rather than age recapitulated said relationship. On the other hand, more recent studies—using parametric mortality laws—found that spontaneous tumour incidence in mice is age‐dependent (Pompei, Polkanov, and Wilson 2001—but see the authors' correction in Harding, Pompei, and Wilson 2012). Reports from other strains (and using non‐parametric statistics) found the same age‐dependent increase in other laboratory rodent strains (Anisimov, Ukraintseva, and Yashin 2005).

Those contrasting results highlight the complexity of using laboratory rodents: even when looking at ‘spontaneous’ tumours, genetically engineered mice are usually preferred to either outbred animals or animals developing truly spontaneous tumours (He et al. 2015). This variance in the genetic background has an influence on the shape of the mathematical relationship between incidence and age.

##### 1.1.1.3 Tumour Incidence at Old Ages: Of Mice and Men

Under the multistage model, as mutations accumulate with age, cancer incidence is supposed to be an increasing function of age. An increasing body of literature shows that this is not the case: when incidence data for all tumour sites (i.e. tissue of origin) are pooled, rates are shown to increase up to 80 years old to then decrease (US data in Thakkar, Villano, and McCarthy (2014); Japan data in Arbeev et al. (2005)). This pattern is also found when tumour sites are individually considered (Harding, Pompei, and Wilson 2011) and when—within a given site of origin—distinct molecular subtypes (Anderson et al. 2006). Consequently, the lifetime risk of being diagnosed first increases with age and then decreases in the oldest individuals (White et al. 2014). In domestic dogs, the number of individuals diagnosed with cancer peaks between 7 and 11 years of age and then decreases in older categories (Rafalko et al. 2023). Similarly, incidence peaks at around 10 years of age to then decrease (Dobson et al. 2002).

In humans, the deceleration of cancer incidence in the oldest ages has been known for decades (Cook, Doll, and Fellingham 1969) and was first explained as a bias (either detection or age‐cohort). A detection bias is unlikely, as reviewed by Anisimov, Ukraintseva, and Yashin (2005). Moreover, Hanson and colleagues showed that the pattern holds even when an age‐period cohort effect is taken into account (Hanson et al. 2015). One last explanation would be that, as the population ages, susceptible people develop malignant tumours until no susceptible individuals remain in the oldest olds (i.e. selective disappearance or frailty hypothesis, Vaupel, Manton, and Stallard 1979) but, as reviewed by Anisimov, Ukraintseva, and Yashin (2005), this is unlikely to be the only explanation in humans.

Whether this deceleration of cancer incidence rate is found in other species is unknown as it would require a study population large enough so that data in the oldest olds are extensive enough to rule out any statistical bias. Conflicting evidence is found in laboratory rodents: the finding of Pompei, Polkanov, and Wilson (2001) that mice cancer mortality would drop to 0 in mice older than 3 years was later corrected: current evidence from that dataset shows that mortality flattens but does not decrease (Harding, Pompei, and Wilson 2012). On the other hand, Anisimov, Ukraintseva, and Yashin (2005) cite two datasets (one for mice and one for laboratory rodents) that seem to indicate that a deceleration could be found. As explained above, the use of genetically engineered laboratory rodents makes it difficult to assess the incidence rate of spontaneous tumours.

Several mechanistic and evolutionary explanations for the deceleration of cancer incidence rates in the oldest old humans have been proposed. First, it has been suggested that—in their original paper—Doll and Armitage used a mathematical approximation that did not include a potential asymptote in the predicted incidence curve. Their model nonetheless remains predictive as they include data up to 75 years old (so before the deceleration). However, other models do predict an asymptote, for example, due to the limited number of susceptible cells (Moolgavkar 2004). Second, it has been empirically shown that cell division rates in 4 human tissues decreased with age (Tomasetti et al. 2019). In other terms, a deceleration of cancer incidence in old age would be the consequence of a decrease in cell turnover with age. Interestingly, in the same study, the same tissues in mice either did not show such a decrease or showed a decrease in a smaller extent. This could explain the discrepancy between humans and laboratory rodents regarding cancer incidence rates in the oldest olds.

The two previous mechanisms could explain the pattern at old ages but fail to explain why an organism ends up with such a limited cell stock or decreased cell turnover and why different species might have evolved those traits or not. In that regard, an abundant body of literature has been highlighting the role of cellular senescence (defined as a stable arrest of the cell cycle, see e.g. Campisi 2018) as a potential defence mechanism against tumourigenesis. Indeed, when becoming senescent, damaged cells stop dividing rather than taking the risk of accumulating even more damage and cellular senescence can be induced by oncogenes (see e.g. Wyld et al. 2020; Schmitt, Wang, and Demaria 2022). An exhaustive review of the literature highlighting the link between cellular senescence and the age‐specific risk of developing cancer is out of the scope of this paper, but two results are interesting to keep in mind. On the one hand, an extension of the Doll‐Armitage model including an extra term to account for cellular senescence has been shown to predict incidence curves more closely than the original model (Pompei and Wilson 2001). This adds to the previous paragraph showing that the mutation‐centric approach to explain cancer incidence lacks the explanation power of models that consider more mechanisms. On the other hand, a recent model showed another cellular mechanism that could be important to consider: apoptosis. This model showed that cellular dynamics could be translated into age‐specific incidence rates that resume the pattern of incidence deceleration with age (Bieuville et al. 2023). This model includes life‐history traits that can also predict whether relying on cellular senescence could be beneficial as a defence mechanism against cancer in a broader range of species. To circle back to the two first mechanisms: cellular senescence—by decreasing the pool of mitotically active cells—can explain the hypothetical asymptote and the empirical decrease in cell turnover.

Overall, this section shows that, for the limited number of species where age‐specific data exist, the Doll‐Armitage accurately predicts the age‐specific risk of cancer in a limited age range. However, this model does not explain juvenile cancer and the discrepancy between its theoretical predictions and empirical observations highlights (i) that there are other mechanistic and evolutionary processes shaping the age‐specific risk of neoplasia (and cancer) and (ii) that those processes can differ between species.

#### 1.1.2 Age and Cancer Types

The evolutionary tumourigenesis model (Nowell 1976) posits that accumulated mutations must be accompanied by stepwise selection and clonal expansion of more competitive cells for tumourigenesis to occur. Additional work also suggests that mutations will be advantageous within a given evolutionary cellular landscape (Rozhok and DeGregori 2015). Because cellular microenvironments are likely to change with age, one can also expect different tissues to have differential risks at a given age or a variable age‐specific risk. Therefore, in this section, we will review what is known about the distribution of cancer types with age.

##### 1.1.2.1 Paediatric and Adult Cancers Have Different Type Distributions

The distributions of cancer types in children and adults support the expectation that different tissues will be most at risk of undergoing carcinogenesis at different ages: the latest GLOBOCAN data (https://gco.iarc.fr/) (International Agency for Research on Cancer n.d.) shows that the most frequent tumour types globally found in 0 to 19‐year‐old children are leukaemia (age‐standardised incidence rate, ASR = 3.2) and tumours of the Central Nervous System (hereafter ‘CNS’, ASR = 1.2). In contrast, the most frequent tumours found in adults are breast (ASR = 70), prostate (ASR = 51.2) and lung (ASR = 37.4).

Mechanistically, this can be explained by the different aetiology between paediatric and adult tumours: paediatric tumours emerge from tissues after periods of quick growth (Hewitt, Weiner, and Simone 2003 and below) or from cells deriving from embryonic layers (Comitani et al. 2023) whereas a majority of adult tumours are carcinomas, developing from epithelial tissues that proliferate extensively. Even when paediatric and adult tumours arise in similar tissues, their genetic alterations have been shown to differ. For instance, paediatric gliomas more frequently feature mutation in histone genes compared to adult gliomas (Mackay et al. 2017) and adult ALL features a chromosomal translocation (‘Philadelphia chromosome’) in 20%–30% of the cases whereas this alteration is found in < 5% of the childhood ALL cases (Koo 2011).

This association between the histological origin and the age‐associated risk of developing cancer is even different in different childhood age ranges. For instance, between 0 and 14 years old, the most frequent tumours are leukaemia whereas between 15 and 19 years old, the CNS tumours are more frequent (datasheet from the USA National Cancer Institute; NCI 2023). Childhood tumours can be divided into three categories: infancy, childhood and puberty tumours (see table 1 in Mora 2012). Without any surprise, this classification shows a clear association between histological origin and spurs of growth. For instance, the most frequent tumours between 0 and 4 are those of the CNS during the period of the fastest CNS growth (see Mora 2012 for a more extensive illustration of the differential risk depending on growth).

##### 1.1.2.2 Adulthood Cancers Are Diagnosed at Different Median Ages, Suggesting Variable Age at Onset

Similarly to paediatric cancers, adult cancer types are characterised by different age‐specific risk depending on the histological origin, as highlighted by different median ages at diagnosis for different tumours. For instance, in the US in 2013, the median age at diagnosis for breast cancer was 61 and 73 for urinary bladder cancer (Karami, Young, and Henson 2007).

Mechanistically, this variation in the mean age at diagnosis has been explained by the difference in the number of stem cell divisions (Tomasetti and Vogelstein 2015), aberrant epigenetic burden in tissues (Klutstein et al. 2017), variation in tissue‐specific mutation rates (Hao, Wang, and Di 2016), exposure to chemical and differential molecular pathways (Schaefer and Serrano 2016) or even different susceptibility of tissues after viral exposure (Okodo et al. 2023; Zapatka et al. 2020).

Evolutionarily, two explanations have been proposed. First, the fitness landscape of the tissue changes with age (Laconi, Marongiu, and DeGregori 2020) and it is likely that those changes will be asynchronous between tissues (Walker and Herndon 2010), between traits (Hayward et al. 2015) or even between individuals (Oh et al. 2023). Second, the different risks for different tissues might correlate with how important those tissues are for survival and reproduction (Thomas et al. 2016; Gidoin et al. 2018). Overall, these potential evolutionary processes are important to consider as half of the driver mutations are thought to occur before the onset of neoplasia (Tomasetti, Vogelstein, and Parmigiani 2013), which shows that the age at diagnosis might not reflect the age‐dependency of carcinogenic processes entirely.

#### 1.1.3 Age, Cancer Prognosis and Cancer Mortality

The link between age and cancer prognosis or mortality is not entirely straightforward: age is often taken as the age at diagnosis rather than the stage at diagnosis. Yet, in humans, the stage is more predictive of the survival prognosis (Hawkes 2019) and the association between age and stage at diagnosis is different for different tumour types (Goodwin et al. 1986), reflecting medical practice that may differ according to the patient's age. Lastly, the age at diagnosis and, therefore, the stage and the survival prognosis are influenced by socio‐economic factors such as the country's GDP (Magrath et al. 2013).

In humans, even if paediatric cancers are rare and overall account for < 1% of all the deaths attributed to cancer across all ages (SEER n.d. data from the USA population, years 2016–2020), cancer accounts for 23% of children's mortality. In contrast, neoplasia accounts for < 5% of juvenile deaths in domestic dogs (Fleming, Creevy, and Promislow 2011). Age‐specific mortality also varies between human paediatric tumours: overall, the mortality rate is higher among the 15–19 yo except in the case of medulloblastoma and neuroblastoma with the mortality rate being higher in 0–14 yo (Saletta, Seng, and Lau 2014). Different tumours can also have drastically different 5‐year survival rates. For instance, ALL has a 79% 5‐year survival rate while AML peaks at 41% (Hewitt, Weiner, and Simone 2003). Looking at the 5‐year survival and later health prospect is important: while immense progress has been made in treating paediatric cancer, survivors experience a decrease in their healthy lifespan expectancy (Force et al. 2019) and are more likely to develop chronic health conditions (Bhakta et al. 2017). From an evolutionary point of view, this is important as surviving paediatric cancer will likely affect not only survival but also reproduction.

Between children and adults, for tumours arising in common tissues, survival can also differ: ALL has a 5‐year survival rate of 79% in children as opposed to 20%–30% in adults under 65 yo (Hewitt, Weiner, and Simone 2003). The same trend is observed for sarcomas (Van der Graaf et al. 2017). The reasons for this discrepancy are multifactorial and depend on both etiological and care factors.

In adult humans, the age‐specific mortality curve follows that of the incidence: the mortality share due to cancer increases up to 75 years old and then decreases in the following age categories (SEER n.d. data from the USA population, years 2016–2020). The same trend is found in domestic dogs with cancer accounting, overall, for 30% of all adult deaths but increasing up until 8–10 years to then decrease (Fleming, Creevy, and Promislow 2011). In contrast, humans and dogs differ in the cumulative share of mortality attributed to cancer as dogs experience larger mortality from neoplasia at ‘equal age’ (see figure 3 in Hoffman et al. 2018).

In the oldest old humans, a recent review of several datasets showed that mortality plateaus in 90 yo and decreases in centenarians when individuals are then more likely to die from other causes (Nolen et al. 2017). This paradoxical observation has led to the hypothesis that the physiology of the oldest leads to less aggressive tumours. One of those physiological aspects would be immunosenescence: as individuals age, their immune efficiency declines and clonal lineages that would not have escaped the immune system in relatively younger patients can expand, making it possible for less aggressive tumours to develop (see e.g. Falci et al. 2013; Lian et al. 2020). Support for this hypothesis has been limited in Humans due to limited data from several tumour types (Van Herck et al. 2021). A second physiological aspect, mentioned above, is the role of the microenvironment. Indeed, it is possible that the physiology of older patients does not permit the extensive angiogenesis and microenvironment remodelling required by fast‐growing tumours with extensive metabolic needs.

Some authors have nuanced the affirmation that all cancers in aged individuals are less aggressive (de Magalhães 2013) but the review from Pavlidis, Stanta, and Audisio (2012) focusing exclusively on cancer in the oldest olds reports three features in support of this affirmation. First, more incidental tumours (i.e., tumours diagnosed during necropsies but not thought to have caused death) were found in the oldest olds. Second, tumours in the oldest olds are less likely to metastasise. As metastasis is often the cause of death, this might explain why neoplasia accounts for 4% of the mortality in centenarians. Lastly, multiple tumours are more frequent in the oldest olds even if mortality from cancer is low. Overall, this suggests that, indeed, features of the aged physiology change the neoplastic processes. This highlights that neoplastic processes might be age‐dependent but that their consequences (e.g. the tumour aggressiveness) also depend on additional age‐specific contexts.

In other species, data are scarce. A study found that two lines of laboratory rodents (one rat and one mice line) allowed to live up to 30 months had cancer mortality rates of around 16% (Miyaishi et al. 2000) but a more thorough review of different strains highlighted the complexity of deciphering the interplay between strain, tumour type and even the administration route of cancer cells to the host (Anisimov 2006): depending on those factors, tumour growth rate and survival can be different or not between different age categories. Another study in flies showed that, for two types of tumour, age at onset was different and only one type could metastasise, also suggesting different age‐specific mortality (providing that, in flies too, metastases cause higher mortality; Salomon and Rob Jackson 2008).

#### 1.1.4 What is Known Apart From Humans and Domesticated Species?

Overall, the previous sections have shown that for the species with extensive data (humans, domestic species and—to a lesser extent—laboratory rodents), there are still some aspects of the age‐related neoplastic risk that remain to be elucidated. But those are not the only unknowns: the question remains for the huge majority of species where extensive age‐specific data do not exist.

For instance, some species such as bats (Wang, Walker, and Poon 2011) or naked mole rats (Liang et al. 2010; Delaney et al. 2016) are thought to have low tumour rates, some others are thought to have co‐evolved defence mechanisms along a large size or a long lifespan (see Seluanov et al. 2018 for a review). Comparative genomic studies also suggest that ageing, tumour incidence and molecular processes ‘coevolve’ so that long‐lived species are better protected (Ricci et al. 2023). However, no data exist to assess whether patterns of age‐specific cancer risk found in a handful of species can be generalised to other species.

Producing those data will prove challenging. First, most of the prevalence data known to date come from captive populations. While informative, those data do not necessarily reflect the dynamics in natural populations where individuals can die from competing causes mostly absent in captivity (e.g. predation). Furthermore, the effects of captivity on life expectancy differ between species. Second, should extensive age‐specific mortality data exist in other species, cancer could remain a rarity and difficult to detect. Lastly, in captivity as in the wild, long‐lived species would need to be followed for a long period of time before any conclusion could be drawn.

### 1.2 Neoplastic and Carcinogenic Processes Are Influenced by Seasons

Seasons regulate the biological activities of most, if not all, organisms, including their constituent cells and inhabitants, from commensal and mutualistic microorganisms to parasitic taxa. Since tumour cells are living entities within their host, it is predicted that they should be influenced by seasonal oscillations as well, and in ways that may differ from normal cells. Additionally, in some species, seasonal changes in behaviour could be affecting or affected by tumourigenic processes and ‘season’ can therefore be understood as a succession of biological cycles such as reproduction (see Box 1 about Tasmanian devils).

BOX 1 **Tasmanian devils as an example of the complex interplay between age, season and immunity shaping the cancer risk.**

Devil facial tumour disease (DFTD) is a unique combination of cancer (defined as an invasive malignant tumour) and contagious disease that has drastically reduced the Tasmanian devil population over the last 40 years (Lazenby et al. 2018). DFTD originated from a Schwann cell in an individual devil and has evolved to spread between individuals as an infectious disease (Murchison et al. 2010). The disease is transmitted by biting, which occurs during food squabbles and is an integral part of their mating behaviour (Pearse and Swift 2006). Previously opened wounds around devils' faces and within their mouths, most likely facilitate disease transmission (Hamede, McCallum, and Jones 2013), although the lack of immune recognition is key to the disease progressing in each new host. Originally, theories suggested the reduced genetic variation between Tasmanian devils leads to a lack of immune recognition (Miller et al. 2011; Cheng et al. 2012). However, this is not the complete story, as DFTD has evolved to downregulate MHC class I, a common strategy of cancers to evade immune detection (Siddle et al. 2013).

Once established, DFTD is fatal for almost all devils. There is evidence of limited immune response by some individuals, with a small number of tumour regressions detected across the population. Current evidence and theories behind tumour regressions show differential expression of key host loci (Margres et al. 2018), the ability to generate tumour‐specific antibodies (Pye et al. 2016), detectable differences in tumour genotypes (Margres et al. 2020) and the possibility of specific tumour microbes (Raven et al. 2024). While the evidence of adaptation is encouraging, a detectable, active immune response to DFTD infection has been difficult to describe. Researchers have speculated that reduced genetic diversity and physiological constraints have limited novel immune responses; however, devils may not invest highly in immune maintenance as they age, evidenced by a decrease in both immune gene expression (Cheng et al. 2017; Raven et al. 2022) and the production of novel T‐cells post‐puberty (Cheng et al. 2019). This immunosenescence may contribute to the success of DFTD, especially if devils, like closely related species, don't invest highly in longevity and tumour suppression, only living relatively short lives (Austad and Fischer 1991). This life history strategy may explain why DFTD evolved successfully twice in devils.

There are likely multiple factors influencing when devils become infected with DFTD and how fast the disease progresses. Mating season, traditionally in late summer, involves increased interaction and fighting between devils (Hamilton et al. 2020), and a period of higher DFTD transmission. For male devils particularly, the stress of mating season can deplete resources (Jones et al. 2008) so this period following mating season may lower male devils' resistance to disease, allowing DFTD a better chance to establish in a new host. In addition, the rate of DFTD progression within individuals could be influenced by the seasonal fluctuations in immune cells (Peck et al. 2015) and immune gene expression (Raven et al. 2022) throughout the year, contributing to differences in tumour growth rates and individual life expectancy.

The linking of health statistics to seasons has been the subject of many studies, particularly in the field of infectious diseases (Altizer et al. 2006; Fisman 2007). For example, it is well established that seasonality can influence host–pathogen interactions in several ways, as seasonal changes often alter variables relevant to infectious dynamics, such as contact rates and social behaviour of hosts, the likelihood of encountering infectious stages in the environment and host immunity (Poulin 2020). Concerning neoplasia, several studies have also attempted to find links between various statistics and seasonality. As tumours are often detected long after the onset of tumourigenesis, it is however more complicated than for infectious diseases (where the time between infection and onset of symptoms is often shorter) to detect correlations with a given season. Also, season is not in itself an independent predictive factor. Multiple variables change with the season, such as light exposure, temperature, feeding requirements, reproductive physiology, pathogens, etc. Therefore, the latitude at which a given study is conducted matters as it will change the values of the possible causal variables (Oh et al. 2010). For example, winter in Norway or Spain corresponds to very different values of the supposed variables of interest, which adds considerable noise to the deciphering of seasonal trends. Current consequences of climate change are also likely to add noise to observed patterns (see for instance Kaffenberger et al. 2017). Finally, spurious correlations may exist. For instance, in humans, there are periods when people allow more time to go for medical examinations, which should lead to peaks in diagnoses unrelated to the actual time when tumourigenesis took place (Bianconi et al. 2016). Additionally, in non‐human species, neoplasia is often diagnosed during necropsies (see e.g. Vincze et al. 2022) which might give an idea of seasonal mortality but that does not necessarily correlate with the influence of season on tumourigenesis.

Although season seems to be a complex and composite variable because of its interactions with multiple factors, there seem to be some interesting trends to consider based on several studies. In this part, we will mainly present correlational studies but given the aforementioned reasons, evolutionary inferences are limited. Concretely, the following topics were explored: (1) the relationship between season and types of tumour diagnosed, (2) the relationship between the season in which certain tumours are diagnosed and patient survival, (3) seasonal variation of cancer mortality and (4) the influence of month of birth on the likelihood of developing certain types of tumour.

Regarding the first issue, there are a number of factors that can vary from season to season and influence the risk of neoplasia, such as sun exposure, on which ultraviolet radiation depends, but also the synthesis of vitamin D, sleep duration, hormonal levels, immunity, etc. Not surprisingly, seasonality in the occurrence of several cancers has been detected, even if consensual conclusions are lacking. Concerning skin cancer, seasonality in the occurrence has been reported several times (see for instance Crocetti and Carli 2005; Ross et al. 1999; Crocetti et al. 2009; Bianconi et al. 2016). While a high incidence rate in autumn could be due to spurious correlations (e.g. more dermatological consultations after the summer holidays, and increased visibility of pigmented skin lesions during this period), it could also depend in part on a delayed effect of UV exposure on tumour progression. Conversely, insufficient exposure to sunlight is an important public health issue that has been proposed to increase the incidence of breast, colorectal and prostate cancer (Alfredsson et al. 2020). In another study based on 2,921,714 breast cancer cases diagnosed across 64 global regions over spans from 2 to 53 years, breast cancer was consistently diagnosed more often in spring and fall, both in the Northern and Southern Hemispheres, regardless of presumable menopausal status. Concerning paediatric cancers, seasonal trends in incidence and diagnosis have been sometimes detected but on average the results are inconsistent (Ross et al. 1999; Nurullah et al. 2018). Thus, largely because of the time lag between actual tumoural initiation and diagnosis, the influence of the seasonal variation remains very complicated to demonstrate.

Regarding the relationship between the season in which certain cancers are diagnosed and patient survival, several studies mentioned a possible link. For instance, Lim et al. (2006) analysed the role of the season of diagnosis and sun exposure in cancer survival for breast, colorectal, lung, prostate and all sites in one million cancer patients in the UK. Cumulative sun exposure in the months prior to diagnosis and season, especially in women, was predictive of subsequent survival. This might indicate that vitamin D metabolites play an important role in cancer survival. Concerning prostate cancer, patients diagnosed during summer and autumn seem to have the best prognosis (Lagunova et al. 2007).

Data regarding seasonal cancer mortality seem to indicate that there are no consistent patterns. Indeed, in humans, mortality (in high latitudes) is higher in winter and cancer mortality during winter is not significantly higher once this is accounted for (Moan et al. 2010). Another study done on 30 years of monthly data in Hungary did find a seasonal pattern in mortality with a peak in August–September but the authors do not mention whether they account for the excess of winter mortality (Virág and Nyári 2019). As explained above, the season diagnosis seems to affect prognosis but the fact that mortality does not consistently display the same pattern suggests that additional factors play a role (Wikén, Andersson, and Radkiewicz 2023).

The developmental origins of health including the season of birth, a surrogate of seasonal variation of environmental exposures, have been linked to a wide variety of later life conditions (e.g. Cecen 2020; Efird and Nielsen 2008; Si et al. 2017), including neoplasia. For example, it has been suggested that the risk of developing lung cancer and squamous cell carcinoma is comparatively lower in people born in winter than in those born in other seasons (Hao et al. 2017). The reasons for this heterogeneity are not clear, but differences in the immune function and the maternal nutritional status during pregnancy may, at least partially, explain this result. According to Park and Kim (2022), men born in winter compared to those born in summer have larger prostate volumes and more prostate cancer, suggesting that the link between season of birth, prostate size and prostate cancer may be due to differences in light exposure in early pregnancy. In China, the risk of developing gastric cancer was comparatively lower for people born in summer than for people born in winter (Xiong et al. 2020). In Tasmania, an increased risk of endometrial cancer has been observed in women born in summer. One proposed explanation is that the longer days and/or more hours of sunlight in the southern states of Australia in summer may suppress melatonin levels in children born in summer and predispose them to cancer as adults (Rowlands et al. 2011). Other studies found a seasonal effect in the monthly birth rates of people who develop colorectal cancer with a disproportionate excess of cancer in those born in September (Francis et al. 2017). Concerning skin cancer, Francis and colleagues found that people born between February and April showed significantly elevated risks of non‐melanoma skin cancer compared with those born in summer and proposed that exposure to ultraviolet radiation in early infancy is associated with an increased risk of neonatal skin tumours. Other studies however failed to detect links (Ljung et al. 2020). For breast cancer, at least two studies have failed to detect a relationship between the month of birth and cancer incidence (Yuen et al. 1994; Hu et al. 1996). No relationship was detected with testicular cancers either (Prener and Carstensen 1990). Regarding paediatric cancers, (Basta et al. 2010) reported a significant sinusoidal variation based on the month of birth for ALL (peak in March), acute non‐lymphocytic leukaemia (peak in September) and astrocytoma (peak in October).

The previous works focused on humans as data allows us to draw correlations and focus on the seasonal variations in environmental conditions. In other animal species, such data either do not exist or will lead to spurious correlations. Additionally, as explained above, it is also important to consider seasonal changes in behaviour and/or physiology. Even if such data is scarce, one can draw predictions about other species from evidence in humans. For instance, seasonal variation in immune efficiency is likely to play a role (for instance, in Tasmanian devils, Box 1). Similarly, in species where reproduction is seasonal, we can expect that any influence of a tumour on the reproductive behaviour of individuals will affect their reproductive output (for instance, in Tasmanian devils, Hamilton et al. 2020). Lastly, as tumourigenesis is tightly linked to metabolism, it is expected that hibernating species (hibernation being characterised by a reduced metabolism) will display a seasonal variation in neoplastic risk. Interestingly, it has been shown that plasma transfusion from a hibernating carp (*Cyprinus carpio*) in mice decreased the tumour size and the number of metastatic subpopulations (Golpich et al. 2022).

### 1.3 Neoplastic and Carcinogenic Processes Are Influenced by the Circadian Clock

The circadian rhythm, also known as the circadian cycle, is a hierarchical multi‐oscillator structure that has an immense impact on the physiology and behaviour of almost all living species. To adapt to and optimally respond to environmental changes in a timely manner, most organisms have developed an internal timekeeping system that allows self‐sustained, oscillating physiological processes that cycle with a ~24 h period (Sancar and Van Gelder 2021). Therefore, one might expect that it could play a role in tumourigenic and carcinogenic processes in pathological contexts where this clock is dysregulated as well as in physiological contexts.

Indeed, clock complex transcriptional‐translational auto‐regulatory networks govern the expression of clock‐controlled regulators as well as genes that have been linked to cancer (Cortés‐Hernández et al. 2020). Although the underpinning molecular mechanisms are not fully elucidated, an increasing number of studies suggest that long‐term deregulation of the circadian rhythm or its gene network can contribute to carcinogenesis (Lee 2021). This abnormal distribution is shown to have an impact on tumourigenesis, tumour growth, metastasis, tumour immune escape and cancer‐related processes such as cell apoptosis and cell proliferation (Xuan et al. 2021). In the following part, we will focus on the role of the circadian clock in pathological and physiological contexts. To our knowledge, no work in non‐human species (except in lab species used to approximate human dynamics) exists so a comparative oncology approach is possible. We will therefore restrict our review to works done in humans.

#### 1.3.1 Cancer Risk as a Function of Night Shift and Sleep Duration

Night‐shift work as well as sleep disorders lead to a misalignment of the body's biological clocks, which has been suggested to be carcinogenic (Sancar and Van Gelder 2021; Cordina‐Duverger et al. 2022). The majority of published studies looked into the link between night‐shift work and breast cancer risk. According to Schernhammer et al., who conducted 10‐year prospective cohort studies among nurses who worked night shifts, nurses who have worked more than 30 years of night shifts are 36% more likely to have breast cancer than those who have never worked night shifts (Schernhammer et al. 2001). Two comprehensive evaluations of night‐shift workers showed increasing evidence of a relationship between increased cancer incidence and all‐cause mortality attributable to circadian rhythm disruption and melatonin suppression (Booker et al. 2018; Wei et al. 2020). Wei, Chen, and Lin recently published a meta‐analysis supporting positive associations among night‐shift work, breast cancer incidence, and cardiovascular mortality (Wei, Chen, and Lin 2022). These findings mirrored their previous meta‐analysis which demonstrated there is not only an increased risk of developing breast cancer and mortality rate, but also an increased risk of breast cancer morbidity (Lin et al. 2015). However, contrary findings in big cohorts involving over 100,000 women in Denmark and the United Kingdom showed no increased risk of breast cancer during night shift work (Vistisen et al. 2017; Jones et al. 2019). This highlights the critical need to consider potential variables, particularly those connected to participants' behaviour and/or socioeconomic position.

The influence of the disruption of the circadian cycle and the carcinogenic effect of being exposed to light have been demonstrated in animal experiments as well. Mice with an inability to regulate their circadian rhythm effectively have an increased cancer risk (Straif et al. 2007; Lee et al. 2010). Chronic jet lag mice also showed a shorter lifespan and accelerated development of liver cancer (Inokawa et al. 2020). Moreover, in a setting where mice were maintained under lighting conditions that modelled the two‐shift system to evaluate the effect of light/dark cycle disruption on metastasis, circadian disruption had little effect on tumour growth while there was a significant increase in the frequency of distant metastasis, implying that night‐shift influences metastasis and prognosis (Numata et al. 2021). More recently, Diamantopoulou et al. showed that the levels of circulating tumour cells (CTCs), pioneers of the metastatic cascade, were increased at night in in vivo breast cancer models as well as in patients (Diamantopoulou et al. 2022). Their result showed that non‐continuous generations of CTCs in the rest phase are more likely to metastasise, which will undoubtedly have major medical implications. However, whether sleep and/or the circadian rhythm are involved in the release of CTCs or the metastatic spread and the specific mechanisms are still unclear (Dauvilliers, Thomas, and Alix‐Panabières 2022). Most recently, using machine learning, rhythmic gene patterns in normal breast tissue and altered rhythms in luminal A breast cancer were identified. Patients with stronger rhythmic gene expression have reduced 5‐year survival rates and increased expression of metastasis‐related genes (Li et al. 2024).

Few studies have looked into the link between night‐shift work and other cancer types, such as prostate, lung, endometrial, oesophageal, colorectal and bladder cancer (Salamanca‐Fernández et al. 2018; Papantoniou et al. 2018; Schernhammer et al. 2013). In addition to the night shift, sleep duration also might have an influence on tumour risk. For instance, short sleep duration is associated with a higher risk of cancer and the formation of aggressive tumours (Soucise et al. 2018). Furthermore, shorter sleep duration was associated with a higher cancer recurrence score (Thompson and Li 2012). Interestingly, non‐nappers had a higher risk of cancer (Ning, Fang, and Zhang 2023). A longer sleep time was shown to lower the risk of breast cancer (Wu et al. 2008). However, a recent categorical meta‐analysis by Chen and colleagues revealed that neither short nor long sleep duration was associated with increased cancer risk. Even though in Asians, short sleep duration increased cancer risk and long sleep duration enhanced the risk of colorectal cancer, these findings were not significant in the dose–response meta‐analysis. This highlighted the need for long‐term randomised controlled trials and well‐designed prospective studies to establish causality and elucidate the mechanism underlying the association between sleep duration and cancer risk (Chen et al. 2018).

#### 1.3.2 Cancer Risk as a Function of Genetics and Post‐Translational Regulation

Circadian rhythms are governed by a set of core clock genes, including CLOCK, BMAL1, PER and CRY, which form feedback loops to control the timing of gene expression (Cortés‐Hernández et al. 2020).

The escape from the discipline of circadian physiology plays a role in tumourigenesis as the homeostasis of cells and tissues is affected. In return, this impacts the cell cycle and division (Soták, Sumová, and Pácha 2014; Lee et al. 2019), leading to uncontrolled and rapid proliferation, heightened metabolic demands, immune evasion, resistance to apoptosis and the creation of an inflammatory milieu that fosters tumour growth. These can be promoted by abnormality in circadian genes or proteins (Kettner, Katchy, and Fu 2014; Yang et al. 2021). These genes play a crucial role in controlling the DNA replication process and orchestrating the timed response to DNA damage through the regulation of cell cycle regulators (Masri and Sassone‐Corsi 2018; Kolinjivadi, Chong, and Ngeow 2021; Gaucher, Montellier, and Sassone‐Corsi 2018). Downregulation of Bmal1 and Clock genes leads to cell cycle arrest and apoptosis (Dong et al. 2019). They can also impact on angiogenesis. For instance, Bmal1 binds to the Vegf promoter to activate it, thereby regulating its rhythmic expression (Ma et al. 2019; Jensen and Cao 2013). Conversely, Per2, Cry2 and Dec2 have contrasting effects on angiogenesis by regulating Vegf expression. Per2, Cry2 and Dec2 peak in mRNA expression during the night, while Vegf expression peaks during the day suggesting timing the administration of antiangiogenic treatments selectively can enhance their effectiveness (Li 2019).

The circadian clock genes govern the activity of both oncogenes and tumour suppressor genes that, in return, exert influence over the core circadian clock genes, contributing to processes related to the neoplastic initiation and progression. For instance, PER1 directly regulates p53‐dependent signals via the ATM and CHK2 kinases to govern the G1 checkpoint when responding to DNA damage (Hernández‐Rosas, López‐Rosas, and Saavedra‐Vélez 2020). Suppression of the clock gene results in an upregulation of the P53 protein, leading to an increase in apoptosis and concurrent cell cycle arrest (Wang et al. 2016). The expression of the c‐MYC oncogene is also under the influence of circadian rhythms with BMAL1 proteins or the REV‐ERBα and RORα pathways involved (Soták, Sumová, and Pácha 2014; Yao et al. 2021). The absence of Cry2 promotes the stability of c‐MYC, while CRY2–FBXL3 collectively orchestrates the degradation of c‐MYC, constraining malignant cellular transformation (Huber et al. 2016). Also, elevated c‐MYC expression inhibits PER1 transactivation, thereby influencing the post‐transcriptional regulation of clock genes (Repouskou & Prombona 2016). These associations in a pathological context strongly suggest that even before the onset of cancer, neoplastic processes are unlikely to occur equally throughout the circadian cycles.

In addition to the impact of circadian cycle genes and protein regulation on tumourigenesis, the epigenetic modification of these elements also influences tumour development. For instance, chromatin remodelling plays a significant role in circadian control, and the reprogramming observed during tumour development appears to involve the circadian clock (Koike et al. 2012; Ripperger and Merrow 2011).

In cancer patients, it has been shown that the relative expression pattern of clock genes differs significantly from the pattern seen in non‐tumour samples (Ye et al. 2018). CLOCK is found to be upregulated in most tumours, while BMAL1, PER1, CRY2, PER3, CRY1, NR1D1 and NR1D2 appear to be downregulated (Ortega‐Campos et al. 2023). PER2 expression also exhibits variation, being overexpressed or downregulated in various tumours (Ortega‐Campos et al. 2023). Despite this diversity, alterations in circadian clock physiology contribute to tumourigenesis in a similar manner and it can be argued that, in return, tumourigenesis is likely altering the circadian cycle.

#### 1.3.3 Influence of the Circadian Rhythm on Immunity and Metabolism in Physiological Context

A large volume of works proved the involvement of the circadian clock in tumour development, either through the knock‐out of circadian genes or through disruption of the circadian rhythm (e.g. Papagiannakopoulos et al. 2016). Preserving the circadian rhythm has a clear preventive role, as exemplified by the liver homeostasis (Kettner et al. 2016). Many other works explored the disrupted circadian rhythms in tumours (e.g. Altman et al. 2015; Battaglin et al. 2021) or the fluctuations of cancerous processes within a 24 h period, leading to the concept of chronotherapy that consists of chronomodulating delivery of anticancer treatments (see the systematic review from Shuboni‐Mulligan et al. 2019). Notably, it has been recently shown that the circadian rhythm influences metastasis through the diurnal CTC generation (see above and the recent review by Diamantopoulou, Gvozdenovic, and Aceto 2023). All those works investigated cancer development either in the context of disrupted circadian rhythms or through the circadian behaviour of cancer cells. Much less is known about the effects of physiological oscillations within a 24‐h period on oncogenesis. In other words, are tumours developing faster in certain periods of the day thanks to oscillations of physiological processes? A few examples in both immunity and metabolism are briefly discussed below.

The immune system is known to be highly time‐of‐day dependent (Wang et al. 2022; Ince et al. 2023). Both the innate and the adaptive immunities are under circadian control. Many molecular mechanisms underlie these oscillations. For instance, dendritic cells (DCs) migrate into skin lymphatics in a circadian manner with a peak during the daytime explained by the rhythmic secretion or expression of several molecules by DCs and lymphatic endothelial cells (Holtkamp et al. 2021). These data suggest that the effectiveness of neoplasia immunosurveillance could fluctuate over a single day. This hypothesis was recently explored by Wang and colleagues using tumour cell engraftment in mice skin and melanoma development (Wang et al. 2023). It was clearly shown that the ensuing tumour size is dependent on the initial time of day of engraftment. This is due to the rhythmic trafficking of DCs to the tumour‐draining lymph nodes that is crucial for the initiation of a response by T cells. As DCs ingest components of cancer cells and present their antigens to T cells in the lymph nodes where a cytotoxic CD8^+^ T cell can recognise them, become activated and gain the ability to destroy any cell that carries a matching antigen on its surface, this rhythmic trafficking of DCs governs the circadian anti‐tumour effects of CD8^+^ T cells. Similarly, circadian regulation of dendritic cell antigen processing also influences T cell responses to vaccination (Cervantes‐Silva et al. 2022).

Among the array of rhythmic physiological processes, the circadian molecular clock also regulates and coordinates metabolic processes, such as glycolysis, the Krebs cycle, gluconeogenesis, mitochondrial function, lipogenesis, lipolysis, amino acids metabolism and nucleotide synthesis (Reinke and Asher 2019). The circadian clock regulates catabolic and anabolic metabolism and the cellular defences to oxidative stress that can arise from nutrient metabolism. Interestingly, mass spectrometry analyses revealed that the abundance of 30%–60% of nucleotides, amino acids, carbohydrates, lipids and cofactors/coenzymes have circadian oscillations (Verlande and Masri 2019) as exemplified in the mouse liver where 50% of detected metabolites oscillate (Krishnaiah et al. 2017). Especially, metabolites such as NAD+, FAD, acetyl‐CoA, SAM/SAH, with important roles in redox biology, mitochondrial processes and histone/nonhistone protein modifications influencing gene expression are rhythmic (Krishnaiah et al. 2017). Oscillations of those involved in epigenetic regulation are involved in transcriptional rhythms. It is entirely possible that these oscillations generate differential behaviour of key cells for tumourigenesis such as stem cells along the day, with periods where they are more prone to transformation thanks to metabolite levels that increase stemness (for instance, increasing NAD+ in the cell supports stemness and pluripotency, see Igarashi et al. 2019).

In summary, much of the influence of circadian oscillations on neoplastic processes in a physiological (non‐disrupted) context remains to be investigated (first and foremost is a broader range of non‐human species). Specific experimental designs will be required to avoid unwanted effects associated with diet, sleep and other sources of perturbation of the circadian cycles.

## 2 Conclusion

This review focuses on the variation in neoplasia risk across different timescales: age, season and round the circadian clock. All three scales highlight that understanding the variation in neoplasia risk along the life cycles of individuals from different species requires the integration of different biological scales, from genes to organisms and their interactions with their environment. Different evolutionary implications in each part also underline the need to integrate mechanistic and distal explanations. Indeed, the risk of developing tumours results from multiple proximate factors, some of which might have been shaped by evolution (e.g. DNA repair).

The first part of the review emphasises that, while age is a risk factor, (clinically defined) cancer is not only a function of mutations accumulating with time. Indeed, tumours also occur before reproductive ages and those paediatric cancers are characterised by entirely different aetiologies and epidemiologies. Additionally, different tissues have different age‐specific risks of developing malignant tumours, suggesting that age‐specific physiological changes or cellular contexts are also important to scrutinise. Moreover, the incidence deceleration at old ages could be explained by trade‐offs between mortality causes—mediated by cellular senescence—making actuarial senescence a byproduct of defence mechanisms against cancer. Lastly, age‐specific incidence has been mostly studied in humans, laboratories and domesticated mammal species. In those species, cancer incidence is (for the most part) characterised by an increase with age. The lack of age‐specific neoplasia risk for a wider array of species prevents definitive conclusions about the universality of that pattern in other animal species. But we already know from some species that do not get (that much) neoplasia that this universality is unlikely. Similarly, to better understand the link between tumourigenesis and senescence, it is crucial to investigate non‐mammal species. Even more so as some of those species may age differently (in shape and pace). For instance, reptiles have been suggested (Chiari, Glaberman, and Lynch 2018) and, among them, testudines seem to be of particular interest given their extremely low rates of senescence (Da Silva et al. 2022).

The second part of the review points out that—regarding the influence of season on neoplasia risk—most studies are (i) correlational and (ii) done in humans. Two main messages are to be taken home from this section. First, tumours take time to develop and the diagnosis season is unlikely to reflect the extent of the neoplastic processes. Rather, early exposure (for instance, to UV light) seems to be of greater importance. Overall, a clearer understanding of the mechanisms leading to neoplasia and the onset of cancer is needed to dismiss spurious correlations. Second, to better understand the link between seasonality and neoplasia risk, it is crucial to focus on species with marked seasonal variations and to investigate potential evolutionary trade‐offs between neoplasia and other physiological functions (e.g. reproduction).

In the last part, the review stresses out—in humans—the influence of the circadian clock in both physiological and pathological contexts. Indeed, numerous signalling pathways are under the control of this clock. As a result, tumourigenic and carcinogenic processes' occurrence varies with the time of day. Dysregulation of those pathways can lead to the rewiring of some cellular functions and, ultimately, to tumourigenesis. Rewired carcinogenic cells, interestingly, remain under the control of the circadian clock. Several therapeutic avenues attempt to harness that feature to tailor more efficient treatment. While data are missing in other species, it has been hypothesised that those therapeutic opportunities could also be of use in veterinary medicine (Farag et al. 2024).

Two core processes spanning the different biological levels implicated in tumourigenesis are recurrently mentioned in these three parts: metabolism and immunity. Indeed, understanding how neoplasia risks vary with age, across seasons and around the circadian rhythm requires understanding the metabolic and immune variations within organisms. Additionally, it is crucial to understand how different metabolism shapes differences in neoplasia **risk** between species. Lastly, the link between neoplastic processes and immunity calls (again) for a deeper understanding of competitive mortality risks. This has evolutionary implications: shaping of the neoplasia risk will depend on other causes of deaths. For instance, if a species has a high probability of dying from infectious diseases, it is unlikely that evolution favours elevated defences against neoplasia and cancer. Overall, this shows that time variations in neoplasia risk will also be better understood if integrated within an eco‐evolutionary framework.

## Conflicts of Interest

F.T. is an Editorial Board member of Evolutionary Applications and a co‐author of this article. To minimise bias, they were excluded from all editorial decision‐making related to the acceptance of this article for publication. The authors declare no conflicts of interest.

## Data Availability

The authors have nothing to report.
